# 77. A case report on MAGIC syndrome

**DOI:** 10.1093/rap/rky034.040

**Published:** 2018-09-20

**Authors:** Joshua Caplan, Jessica Lee Siew Hua, Priyanka Chandratre

**Affiliations:** Rheumatology, Sandwell and West Birmingham Hospitals, Birmingham, UNITED KINGDOM

**Introduction:** MAGIC syndrome (mouth and genital ulcers with inflamed cartilage syndrome) is a systemic condition consisting of an overlap of Behçet's disease (BD) and relapsing polychondritis (RP). We present the case of a 40 year old woman with MAGIC syndrome who developed a saccular aneurysm of the aortic arch as well as right femoral and popliteal aneurysms, ultimately resulting in emergency above knee amputation. We also discuss the development of these complications despite the patient being on systemic immunosuppressive therapies (azathioprine, thalidomide, infliximab, cyclophosphamide and tocilizumab).

**Case description:** A 34 year old female was referred to City Hospital Birmingham’s Behçet's Syndrome Centre of Excellence in September 2012. This was following an 18 year history of BD and a six year history of RP. The clinical features of these two conditions led to the diagnosis of MAGIC syndrome. She was referred to City Hospital as her symptoms were not controlled despite attempts with multiple different immunosuppressive therapies (azathioprine, thalidomide, ciclosporine, and prednisolone). Examination at that time revealed evidence of active synovitis in the left hand metacarpophalangeal joints, both wrists and both mid-tarsal joints. Ophthalmology assessment revealed bilateral scleral thinning with evidence of partially suppressed scleritis. A decision was made to start infliximab, at a dose of 5mg/kg, at intervals of 0, 2 and six weeks and eight-weekly thereafter. Bloods showed: CRP 98mg/L; ESR 99mm/hr; normal immunoglobulins apart from slightly reduced IgG at 4.9 g/L; Hb 13.1g/L with raised MCV 101fL. Other bloods were unremarkable including liver function tests, rheumatoid factor, complements, anti-nuclear antibody, anti-neutrophil cytoplasmic antibodies, and protein strip. In October 2012, before receiving her 2nd dose of infliximab, the patient reported shortness of breath. This was associated with a reduced exercise tolerance and production of green sputum. Chest x-ray showed extensive air space shadowing bilaterally, and a large area of consolidation in the right mid zone. Given the immunosuppressive medications the patient had previously received, opportunistic infections were considered. High resolution CT showed bilateral patchy ground glass changes through all lung zones. Bronchoscopy was normal and viral polymerase chain reaction (PCR) and mycoplasma were negative. A diagnosis of *pneumocystis jirovecii*(PJP) (HIV negative) was made by PCR. Azathioprine and ciclosporine were stopped and treatment of PJP with septrin was commenced. Following the completion of her treatment for PJP, our patient continued her infliximab regime and was restarted on azathioprine. She remained on septrin prophylaxis and acyclovir was also commenced prophylactically following an episode of herpes zoster infection. She tolerated these treatments well with no further complications from the infliximab. In May 2013, the patient presented acutely with right leg pain. CT angiogram showed an extremely large fusiform aneurysm of the proximal right popliteal artery which measured 9cm in craniocaudal diameter anda number of scattered patchy areas of bone sclerosis in the right femur and tibia which were in keeping with bone infarcts. A right sided endovascular popliteal sheath graft was subsequently inserted and the patient was commenced on dual antiplatelet therapy. During admission, the patient was commenced on her first dose of intravenous (IV) cyclophosphamide (15mg/kg) and given two doses of IV methylprednisolone. The patient subsequently developed a right below knee deep vein thrombosis (DVT). A decision was made not to anti-coagulate the patient. Throughout 2013, she continued to receive IV cyclophosphamide on a three-weekly basis. The intervals were shortened to two-weekly during episodes of poor disease control.In August 2013, she was found to have ischaemia of the right 1st and 2nd toes. Following further investigation with Doppler ultrasound she was found to have a femoral DVT. The patient’s dual antiplatelet regime was stopped and she was subsequently commenced on enoxaparin. In September 2013, the patient developed a rupture of a right common femoral artery aneurysm requiring emergency repair with a Dacron graft in Guy’s and St Thomas’ Foundation Trust. Histopathology showed vasculitis within the arterial wall. Our patient continued to experience problems with distal wound necrosis in the groin. In February 2014, a repeat CT angiogram showed stenotic lesions in both the right groin graft and popliteal stent. Following discussion in the multidisciplinary team meeting, it was decided that angioplasty was needed for both lesions. This was carried out with good results. In June 2014, the patient, now 36 years old, again developed shortness of breath. As part of her investigations a CT thorax was performed. The CT scan showed a large saccular aneurysm of the brachiocephalic artery, which appeared to extendto the level of the bifurcation of the right subclavian and common carotid arteries. There was also a 4.4cm saccular aneurysm arising from the inferior surface of the arch at the level of the left subclavian artery. She underwent replacement of the aortic arch with a branched graft and frozen elephant trunk procedure with the branches of the arch graft going to the left common, right carotid, and the right subclavian arteries. An important and unexpected finding at the time of surgery was a large 3-3.5cm right coronary artery (RCA) aneurysm. This was considered high risk for rupture and required immediate treatment. The aneurysm was ligated proximally and distally and a saphenous vein graft was constructed to the posterior descending RCA. The patient subsequently developed right ventricular failure and required extra-corporeal membrane oxygenation (ECMO) for cardiovascular support. This resulted in a prolonged ICU stay. At her post-operative appointment in October 2014, the patient was mobilising with crutches and was able to stand and walk short distances independently. However, in December 2014, she presented with acute limb ischaemia and required an emergency right sided above knee amputation. In March 2015, it was decided that the patient would start on tocilizumab. By 2016, the patient’s disease was clinically in remission. However, towards the end of 2016, the patient experienced a left-sided upper lobe collapse with lesions at the left lung hilum. Bronchoscopy showed RP causing local bronchial stenosis. A repeat CT scan two weeks later, following a course of steroids, showed a resolution of the lobar collapse and lung lesions. As of 2018, the patient remains on tocilizumab. Her patient index score for BD is 1 and her transformed index score is 3, which indicates low disease activity. She reports to be happy with regard to her disease activity. Clinically our patient’s disease is stable currently, however, it remains precarious as she is at high risk of developing complications from her conditions and as a result of immunosuppressive therapy.

**Discussion:** The number of reported cases of MAGIC syndrome are far and few between. Even at City Hospital Birmingham’s Behçet's Syndrome Centre, the number of cases of MAGIC syndrome is low. Therefore, further research and patient observation is needed for us to fully establish our understanding of MAGIC syndrome.

**Key Learning Points:** MAGIC syndrome is a complex syndrome that requires consideration and a high index of suspicion in patients with BD who present with nasal, tracheal, or auricular symptoms, which may indicate RP. Patients with MAGIC syndrome can develop complications of BD and RP despite systemic immunosuppression. The balance between continual increases of immunosuppression for clinical benefit versus the risk of side effects can be extremely challenging. Therefore, a high degree of suspicion must be employed when reviewing patients with MAGIC syndrome to ensure that new symptoms from the patient’s disease or from the side effects of immunosuppression are not missed. Complex rheumatological conditions such as MAGIC syndrome present clinicians with challenges when developing management strategies. A personalised and patient-tailored approach is required before and during treatment, with an ability to adapt to what can be a constantly evolving clinical picture. Educating patients about their disease and how to manage day-to-day aspects of their condition is a fundamental part of management which develops their role as an active informed participant in their care. This is one of the major aspects of the multidisciplinary approach at the Behçet's Syndrome Centres of Excellence. This holistic approach helps patients understand their condition and access timely and appropriate care.

**Disclosure: J. Caplan:** None. **J. Lee Siew Hua:** None. **P. Chandratre:** None.

